# Effect of Carbon Black and Hybrid Steel-Polypropylene Fiber on the Mechanical and Self-Sensing Characteristics of Concrete Considering Different Coarse Aggregates’ Sizes

**DOI:** 10.3390/ma14237455

**Published:** 2021-12-04

**Authors:** Shakeel Ahmed, Abasal Hussain, Zahoor Hussain, Zhang Pu, Krzysztof Adam Ostrowski, Rafał Walczak

**Affiliations:** 1 State Key Laboratory of Coastal and Offshore Engineering, Dalian University of Technology, Dalian 116024, China; sa222475@outlook.com; 2 Engineering Research Center of Fiber Composite Building Materials and Structure, Ministry of Education, Zhengzhou University, Zhengzhou 450001, China; zahoorhussain@ssuet.edu.pk (Z.H.); zhp1243@163.com (Z.P.); 3 Faculty of Civil Engineering, Cracow University of Technology, 31-155 Cracow, Poland; rafal.walczak@pk.edu.pl

**Keywords:** concrete beam, coarse aggregate, steel fiber, polypropylene fiber, carbon black, conductive concrete, FCR-COD curves, crack monitoring

## Abstract

The effect of combining filler (carbon black) and fibrous materials (steel fiber and polypropylene fiber) with various sizes of coarse particles on the post-cracking behavior of conductive concrete was investigated in this study. Steel fibers (SF) and carbon black (CB) were added as monophasic, diphasic, and triphasic materials in the concrete to enhance the conductive properties of reinforced concrete. Polypropylene fiber (PP) was also added to steel fiber and carbon to improve the post-cracking behavior of concrete beams. This research mainly focused on the effects of macro fibers on toughness parameters and energy absorption capacity, as well as enhancing the self-sensing of multiple cracks and post-cracking behavior. Fractional changes in resistance and crack opening displacement (COD-FCR) and the relationship of load-deflection-FCR with different coarse aggregates of (5–10 mm and 15–20 mm) sizes were investigated, and the law of resistance signal changes with single and multiple cracking through load-time-FCR curves was explored. Results indicated that the smaller size coarse aggregates (5–10 mm) showed higher compressive strength: up to 8.3% and 14.83% with diphasic (SF + CB), respectively. The flexural strength of PC-10 increased 22.60 and 51.2%, respectively, with and without fibers, compared to PC-20. The diphasic and triphasic conductive material with the smaller size of aggregates (5–10 mm) increased the FCR values up to 38.95% and 42.21%, respectively, as compared to those of greater size coarse aggregates (15–20 mm). The hybrid uses of fibrous and filler materials improved post-cracking behavior as well as the self-sensing ability of reinforced concrete.

## 1. Introduction

Structural health monitoring (SHM) is a topic of growing interest in the field of civil engineering, which improves structural safety and enhances the maintenance service intervals of concrete structures under condition-based maintenance. Different cracks and damages occur during the service life of concrete structures through different loading conditions. It was deemed important to investigate the deterioration of concrete structures in time to overcome cracks and damage. Damage detection and crack sensing are typically executed using different sensors (i.e., piezoelectric, piezoresistive, and fiber optic sensors) [1,2,3]. Nevertheless, these sensors have limited applications due to their low durability, high cost, lack of spatial resolution, and limited sensing ability. A new method was developed for self-sensing cracks and damage in concrete structures. In the elastic region, strain creates reversible variation in electrical resistance and the cracks generate irreversible variations in electrical resistance [4,5,6]. A new strain/stress sensing technology was established based on electrically short fiber conducting pull-out technology, which attends to insignificant and reversible crack propagation [7]. Cracking and damage occurrences can be monitored with conductive material inside the concrete structure using electrical resistance measurements of its own strain and stress [8,9]. Over the last few decades, some researchers have classified the expediency of cement-based composite sensors for monitoring the flexural cracks and tensile stresses and inspected for compressive strain with different types of conductive materials [6,10,11,12,13,14,15]. Conductive concrete exposed significantly excessive mechanical strength and high electrical conductivity when compared with conventional concrete [16]. Based on conductive concrete mixture proportioning, various conductive materials (steel fiber, carbon fiber, carbon nanotube, carbon black, nickel powder, etc.) were added and/or used to replace coarse aggregates and compared with conventional concrete [17,18,19,20].

Some conductive materials, such as steel fiber (SF), carbon fiber (CF) and carbon black (CB), were incorporated into the concrete structure, which improved the conductive properties and mechanical properties before and after cracks occurred. Ding et al. [21] reported that, after the appearance of the first crack, macro steel fiber was able to restrain crack widening and enhance resistance against cracking, improving the toughness of concrete beam. Jansson et al. stated that it also increased load-deflection behavior and excessively bridged the conductive path between the cracked concrete beam matrix [22]. The electrical properties of concrete are decreased by the appearance of cracks in self-sensing materials. Steel fibers can bridge these cracks and help to reduce resistance and the rapid spreading of microcracks. However, the hybrid use of different fibers can help to increase the performance of members rather than the specific individual fiber [23,24,25,26,27,28]. The combination of different fibers is known as hybridization, including, for example, steel fiber and polypropylene fiber. During hybridization, the first type of fiber (steel fiber) gives more strength, stiffness, ultimate strength, strain sensing capability and adequate first cracking growth, while the other type of fiber (polypropylene fiber) restricts microcracks and increases the toughness parameters into the post-crack zone in hybrid fiber-reinforced concrete. This phenomenon is also called synergy [29,30]. The hybrid combination of steel fiber and polypropylene fibers can increase post-cracking behavior, flexural strength, modulus of elasticity, post-peak ductility and higher stiffness of FRC [31]. The polypropylene fiber can reduce the quantity and size of micropores in the reinforced concrete and decrease porosity around the aggregates at ITZ [32]. Compared to mono use of SF, the flexural toughness, flexural strength, load-bearing capacity, and modulus of elasticity are higher in combined steel and polypropylene fibers [33,34]. It is due to this that the polypropylene fiber grants resistance against nonloading cracks in reinforced concrete. It is also used as a base material with steel fibers to increase toughness parameters, deflection hardening behavior and restrict microcrack propagation [35,36].

Due to the formation of effective conductive pathways, the electrically conductive fibers and fillers like steel fiber (SF) and carbon black (CB) are more efficient admixtures for enhancing the electrical conductivity in reinforced concrete [37,38]. Carbon black-based cementitious composites showed excellent durable self-sensing abilities. The CB structure contained nanoparticles that decreased the air voids in concrete and helped to improve the conductive path as a filler material with fibers [39]. Fiber-type additives expose more electrical conductivity due to their high aspect ratio. As filler material, nanocarbon black is priced low and, with its excellent electrical conductance, could be used as an optimal admixture for improving the electrical conductivity of concrete [21]. The sensing ability of cementitious composites could be increased by using carbon black as a filler material [12]. However, the filler materials cannot continue the conductive path after the first crack appears and crack localization of reinforced concrete occurs [40,41]. Therefore, fibrous conductive materials, such as steel fiber and carbon fiber, provide extensive self-sensing properties in the concrete structure and improve post-cracking behavior. The combined use of CB and macro steel fiber improved the conductivity of concrete using the electrical properties of carbon black and the conductive pathways of fibers. Additionally, the existence of nanocarbon black may increase the toughness, electrical conductivity, and mechanical properties and decrease concrete pore sizes [17]. Monteiro [42] investigated the properties of cement-based composites with the inclusion of carbon black particles. Results concluded that the monitoring resistivity of carbon black was increased, and the resistivity of CB based composite was decreased with the increase in volume fraction.

In the sensing mechanism, the larger coarse aggregate size extensively affects the conductive pathway, as it hinders the transportation of electrical charges from one conductive body to another. The maximum coarse aggregate size should be no more than 0.5 times the fiber length [43]. When the SF length to coarse aggregates’ maximum size increases, the flexural strength ratio also increases. It continuously increased with the maximum coarse aggregate size (up to 20 mm) and then reduced [44]. The mechanical properties of steel FRC were also influenced by the addition of larger coarse aggregates. These properties include flexural strength, compression strength, and splitting tensile strength. Furthermore, mechanical properties are also improved by small aggregate size. The smaller coarse aggregates reduced the number of videos as compared to the greater number of coarse aggregates, which ultimately improved the mechanical properties. Smaller aggregate sizes have a higher surface area, resulting in strong bonding between the fiber and matrix, which ultimately leads to higher flexural toughness parameters. The results are consistent with the studies by Fazli et al. [45] and Oraimi et al. [46].

Therefore, considering all the above arguments and reasons, the main objective of this work was to investigate the hybrid effects of different fibrous and filler materials with different coarse aggregate sizes on the mechanical properties, post-cracking behavior and conductive properties of the flexural member. Different studies have been done with combinations of steel fiber and carbon black with the same size coarse aggregates; however, the effects of different coarse aggregate sizes of hybrid fiber and filler materials on crack sensing and post-cracking behavior has not yet been studied, to the best knowledge of the authors. Additionally, insofar as the authors could discern, no research has been conducted on crack sensing in concrete with different sizes of coarse aggregates in combinations of hybrid fibers. In this work, the self-sensing effects of two different-sized coarse aggregates (in combination with steel fiber, polypropylene fiber and carbon black) were examined. This study also investigated the load-deflection behavior, toughness parameters, energy absorption capacity, crack opening displacement and fractional change in resistance (COD-FCR) behavior of fiber-reinforced concrete beams with two different sizes of (5–10 mm and 15–20 mm) coarse aggregates.

## 2. Research Significance

Fiber-reinforced concrete is increasingly used in various types of engineering structures. Thus, the development of this material is of great practical importance. Therefore, the advancement of FRC is a relevant topic discussed in many research centers. In addition, the development of self-sensing properties in fiber-reinforced concrete is an important issue for structural health monitoring purposes.

Various FRC research with combinations of steel fiber and carbon black have been conducted around the world. However, the effects of the coarse aggregate size on post-cracking behavior and crack self-sensing in concrete have not been sufficiently explored.

The main objective of this paper was to investigate the hybrid effects of different fibrous and filler materials with different coarse aggregate sizes on the mechanical properties and conductive properties of the flexural member. The novelty of this study was the analysis of the coarse aggregate size effects on crack sensing and post-cracking behavior in distinct types of FRC beams.

## 3. Experimental Procedure

### 3.1. Mix Design of the Concrete Matrix

The basic mix design was prepared for C30 concrete, and the w/b ratio used during this study was 0.5, which was finalized after the detailed calculation and series of the experimental investigations in [11,13]. The concrete specimens were prepared with a binder of Portland cement (P. O42.5R). The cement had a specific gravity of 3.15. Quartz was used as fine and coarse sand taken from a natural river with a 2.51 fineness module between 0–5 mm sizes. Crushed limestone coarse aggregates with two different sizes were used. The size of coarse aggregates varied from 5–10 mm to 15–20 mm (in the second type of aggregate used). Superplasticizer was used to ensure appropriate workability, to obtain high early and final strength and to increase durability. The amount of the admixture modifying the rheology of the concrete mixture was 0.5% of the binder weight (cement + fly ash).

### 3.2. Raw Materials

**Figure 1:**
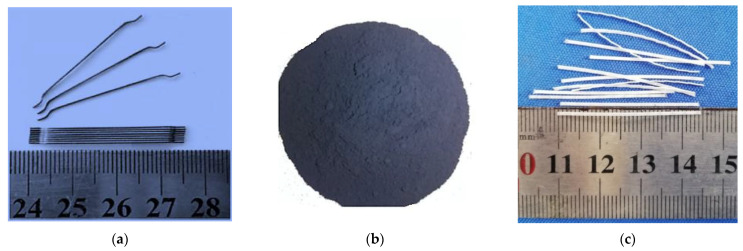
Dimension and shape of (**a**) steel fibers (**b**) nanocarbon black (**c**) polypropylene fibers.

As a fibrous material, the mechanical properties and conductivity of reinforced concrete were increased by the addition of steel fiber (SF), carbon black (CB) and polypropylene (PP) fibers. Figure 1 and Table 1 describe the description of the fibrous and filler materials details. A 35 mm length of steel fiber with a 0.55 mm diameter was used. It had a volume resistivity of approximately 10^−5^ Ω·cm and a 7.85 g/cm^3^ density, as seen in Figure 1a. The slenderness of the steel fiber was 64.

The volume resistivity of nanocarbon black was 2.3 Ω·cm, the density was 0.5 g/cm^3^ and the maximum size containing 30–90 nm was used during this study, as shown in Figure 1b. Polypropylene fiber (Figure 1c) with a 35 mm length and density of 0.9 g/cm^3^ was used. Table 2 shows the relationships between different concrete specimens, i.e., conductive (SF, CB) and nonconductive (PP) fibers with different volume content. The range of volume resistivity varied between 2 × 10^5^ and 10 × 10^5^ Ω·cm for dried concrete without conductive admixtures. For electrically conductive material, the electrical resistivity varied between 1 × 10^3^ to 6 × 10^3^ Ω·cm for dried concrete.

### 3.3. Samples and Setup Description

A maximum of 10 concrete mixtures were prepared during these experiments for compressive strength and flexural tests under bending. During the casting of concrete mixtures, different fiber materials with water were added to the drum and the mixture was rotated for 3 min continuously.

The fresh concrete was cast into cube forms (100 mm × 100 mm × 100 mm) in order to determine the compressive strength, and the flexural test was performed on 100 mm × 100 mm × 400 mm prism beams. All tests were performed after 28 days of concrete maturation. After mixing, the properties of the fresh concrete (e.g., slump value and air-entrained testing) were measured during casting. The fresh concrete was cast in plastic molds after uniform, even mixing and then put into a vibrating table for proper compaction. After placing the molds, the samples were covered with plastic sheets and kept at room temperature (26 °C ± 1 °C) for 24 h. The samples were demolded after 24 h of casting and placed in a curing room at 27 °C ± 1 °C with relative humidity 97% ± 1% until testing (28 days). Four conductive tape adherents were adjusted, designated A, B, C and D as electrical contacts, respectively. Four electrode-AC methods were used to measure the FCR of concrete samples. The distance between contacts A and D was 190 mm and was made for passing the (100 Hz) alternative current. The distance between contacts B and C was 100 mm, which served to measure the voltages. The voltage between the interior contacts B and C was controlled by V_1_ voltmeter equipment.

The voltage of the fixed resistor (*R_f_*) was measured using the V_2._ According to the electrical resistance, the current was determined by calculating the voltage drop over the fitted resistor inside the contacts of beams using Ohm’s law. The mechanical properties and the relationship of load-time-FCR, load-deflection-FCR and COD-FCR were explored in this study. This study also examined the mechanical properties and relationship between load-time-FCR, Load-deflection-FCR, and COD-FCR, respectively. The 12 V DC power supply was used as an external electrode and an inner electrode was used as a fixed resistor (*R_f_*) 5 K. Equation (1) indicates its initial value with time, showing how much resistance changed with fractional changes in resistance.

(1) $$\mathrm{FCR}\;\left(\%\right)=\frac{Rs\;\left(t\right)-Rs\left(to\right)}{Rs\;\left(to\right)}$$

For all concrete mixes, the fresh properties were measured, including reference concrete. Concrete cube samples with 100 mm size were prepared to measure the compressive strength of different conductive and nonconductive concrete mixes. After 28 days, the concrete cube specimens were removed from the curing room and tested for compressive strength.

### 3.4. Flexural Test

**Figure 2:**
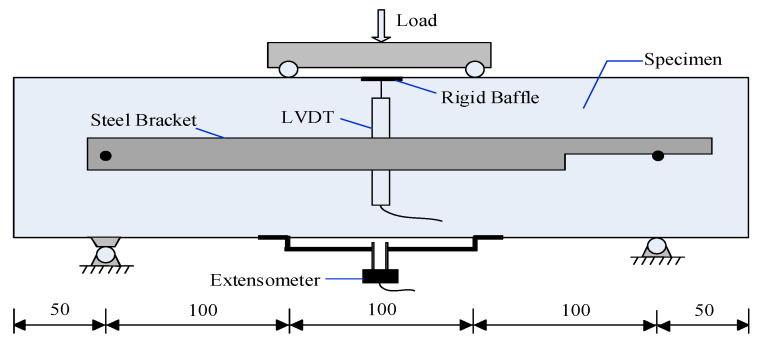
Schematic diagram for bending loading experimental set-up.

The flexural beams were tested with different types of fibers and two different sizes of coarse aggregates over a span of 300 mm according to ASTM C 1609 [47]. A hydraulic servo testing machine was used to perform the four-point bending test on all types of concrete specimens. Two linear variable differential transformers (LVDTs) were fixed on the opposite sides of two beams to measure the deflection behavior. Subsequently, a uniaxial load was applied monotonically with the deformation rate of 0.2 mm/min on the beams until a specified deflection reached 5 mm [48]. The setup of load-deflection curves of concrete beams under bending is also described in Figure 2. An extensometer was fixed under the tension side of beams at the mid-span to monitor the crack opening displacement, as shown in Figure 2. The real-time data was collected using the IMC intelligence data collecting system.

## 4. Results and Discussion

### 4.1. Effect of Size of Coarse Aggregates and Fibers on Compressive and Flexural Strength

The mechanical characterization and evaluation of compressive strength were described in compression test results. Experimental results indicated that the concrete specimens were not overly influenced by the inclusion of steel fiber, polypropylene fiber or carbon black. By including carbon black and steel fibers, the compression strength increased due to the nano-filling effects of carbon black. It improved the microstructure of reinforced concrete and reduced a large number of pores in the reinforced concrete matrix [13]. The compressive strength was measured by an average of three specimens of each mixture at the end of 28 days, as shown in Table 3. The smaller (5–10 mm) coarse aggregates showed higher compression strength values than larger (15–20 mm) coarse aggregates. The maximum increment was observed up to 13.48% and 14.83%, respectively, with SF70-10 kg/m^3^ and SF70-CB4-10 kg/m^3^ volume content in case of 5–10 mm size coarse aggregates compared to reference concrete. In the case of 20 mm size aggregates, the maximum compression strength was increased by 6.75% and 14.2%, respectively, with SF70-20 kg/m^3^ and SF70-CB4-20 kg/m^3^ volume content of reference concrete. This increment was due to the synergistic influence of carbon black filling the microscopic pores in the concrete and decreasing the larger voids between adjacent concrete matrices [11,38]. The compression strength of SF70-10 and SF70-CB4-10 (smaller size coarse aggregates 5–10 mm) increased by 6.70% and 9.01%, respectively, compared to that of (greater size coarse aggregates 15–20 mm) SF70-20 and SF70-CB4-20. The hybrid combination of steel fiber with polypropylene fibers in diphasic admixtures showed a minor reduction: up to 4.45% and 6.21%, in both cases of 10 mm and 20 mm size aggregates compared to plain concrete.

The flexural strength (σ_u_) levels of different beams were investigated according to the international union of laboratories and experts in construction materials, systems, and structures (RILEM) with the addition of conductive fillers and fibers [48]. The inclusion of various fibers and NCB in plain concrete significantly increased the flexural strength. The comparison of flexural strength values with the PC beam values is shown in Table 3, with two coarse aggregates of different sizes. The flexural strength of SF70-10 (beam with SF 70 kg/m^3^ and 5–10 mm size aggregates) and SF70-20 (beam with 70 kg/m^3^ and 15–20 mm size aggregates) increased up to 16.84% and 22.60%, respectively, as compared with PC-10 (plain concrete with 5–10 mm size aggregates) and PC-20 (plain concrete with 15–20 mm size aggregates). The flexural strength (σ_u_) of SF70-CB4-10 and SF70-CB4-20 (beam with SF 70 kg/m^3^, NCB 4 kg/m^3^, and aggregates with 5–10 mm and 15–20 mm size) diphasic conductive material increased up to 35.75% and 40.46%, respectively, compared to PC-10 and PC-20. The flexural strength (σ_u_) of SF70-PP2-10 and SF70-PP2-20 (beam with SF 70 kg/m^3^, PP 2 kg/m^3^, and aggregates with 5–10 mm and 15–20 mm size) increased up to 26.20% and 30.37%, respectively, compared PC-10 and PC-20. The concrete with triphasic conductive material SF70-PP2-CB4-10 and SF70-PP2-CB4-20 (beam with SF 70 kg/m^3^, PP 2 kg/m^3^, CB 4 kg/m^3^ and aggregates with 5–10 mm and 15–20 mm size) increased 51.2% and 33.087%, respectively, compared to PC-10 and PC-20. The influence of NCB and polypropylene fiber was greater on the flexural strength in both diphasic and triphasic admixtures compared to mono SF 70 kg/m^3^. The flexural strength of SF70-CB4-10 increased up to 13.76% compared to SF70-10 and SF70-CB4-20 increased up to 19.65% compared to SF70-20. These results demonstrated that the σ_u_ improved with the addition of nanocarbon black.

In the case of diphasic materials, the σ_u_ was also greater compared to SF70 kg/m^3^. The flexural strength of SF70-PP2-CB4-10 increased 26.67% and SF70-PP2-CB4-20 is increased 13.54% as compared to SF70-10 and SF70-20, respectively, with the addition of PP and NCB. As shown in Table 3, the flexural strength of aggregates of 5–10 mm in length in all cases was greater than that of aggregates 15–20 mm in size. The SF70-PP2-CB4-10 showed a maximum flexural strength increase of 18.31% as compared to SF70-PP2-CB4-20. Reducing the flexural strength of larger size aggregates was possible because the larger size coarse aggregates also had the larger interfacial transition zone (ITZ), leading to a weaker binder zone around the aggregates and increasing the chance of cracking after loading.

### 4.2. Effect of Different Fibers and Coarse Aggregates Size on the Post-Peak Behaviour of Concrete

The experimental investigation was carried out through the RILEM equation on flexural toughness, post-peak behavior, flexural strength, energy absorption capacity/(*D^f^_BZ_*) and equivalent flexural strength/(*f_eq_*) [48]. Toughness parameters and energy absorption capacities of fiber-reinforced concrete were measured according to Equation (2).

(2) $$D_{BZ}=\int_{0}^{\delta i}F\left(x\right)dx$$

**Figure 3:**
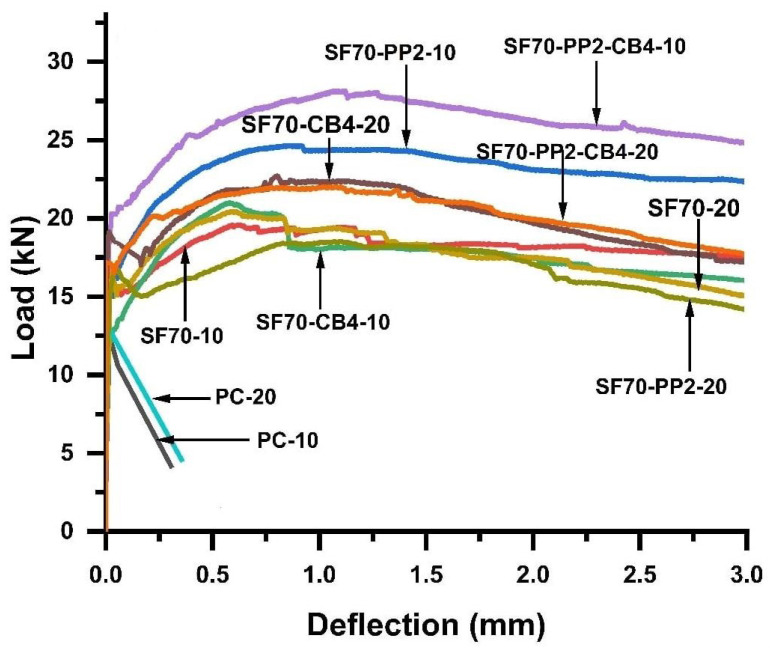
Flexural load-deflection curves of 5–10 mm coarse aggregates and 15–20 mm coarse aggregates.

Table 3 shows the roughness parameters and post-cracking behavior of reinforced concrete with coarse aggregates of two different sizes. From Figure 3, the bending toughness parameters (energy absorption capacity/*D^f^_BZ_*_2,3_ and equivalent flexural strength/*f_eq_*_2,3_) were measured through a load-deflection diagram.

From Figure 3, it can be observed that the behavior of plain concrete samples was linear up to the peak load, after which it abruptly separated into two parts, after first cracking due to the brittle failure. On the contrary, the specimens containing hybrid fibers showed a trilinear variation with both types of aggregates. They showed an extensive cracking pattern over the peak load and initial crack and clearly differed from ordinary concrete specimens. Load decay occurred after the appearance of the first peak load (P_1_), as it depended on the volume fraction of fibers on the crack surface of the concrete. As the microcracks increased in size (macrocracks), the fibers provided a more active path in bridging the crack action. Figure 3 shows the load-deflection behavior of both (5–10 mm and 15–20 mm) types of aggregates during the flexural bending test. The smaller coarse aggregates (5–10 mm) with diphasic and triphasic admixtures showed a higher flexural strength and higher load-deflection behavior compared to that of greater size (15–20 mm) coarse aggregates. Toughness parameters such as energy absorption capacity, flexural equivalent strength, deflection hardening behavior were also increased compared to plain concrete subjected to a four-point bending test. Compared to SF70-10, with 70 kg/m^3^ steel fibers, equivalent flexural strength *f_eq_*_2,3_ and energy absorption capacity *D^f^_BZ_*_2,3_ increased with diphasic and triphasic admixtures. As compared SF70-10, the equivalent flexural strength/*f_eq_*_3_ and the energy absorption capacity/*D^f^_BZ_*_.3_ of SF-CB4-10, SF70-PP2-10, and SF70-PP2-CB4-10 increased by 21.24%, 35.05% and 43.69%, respectively. The volume content of triphasic admixture (SF, PP and CB) contributed substantially to the post-peak behavior (Table 3). This was due to the fact that polypropylene fibers are able bridge microcracks thanks to their lower tensile strength and modulus of elasticity. Additionally, steel fibers can reduce crack widening, delaying crack propagation from micro- to macrocracks, and nanocarbon black reduces large pores due to the nano-filling effect. Comparing the energy absorption capacity and equivalent flexural strength of 20 mm size aggregates to 5–10 mm coarse aggregates (SF70-10, SF70-CB4-10, SF70-PP2-10, SF70-PP2-CB4-10) showed increases of 4.64%, 23.47%, 44.83% and 24.43%, respectively, compared to SF70-20, SF70-CB4-20, SF70-PP2-20 and SF70-PP2-CB4-20. The smaller coarse aggregates reduced the number of videos compared to the greater number of coarse aggregates, which ultimately improved flexural toughness parameters. As predicted, smaller aggregate sizes had a higher surface area, resulting in strong bonding between the fiber and matrix, which ultimately led to higher flexural toughness parameters.

However, when the size of the coarse aggregate exceeded 5–10 mm, entering the 15–20 mm range, the larger coarse aggregate size showed the weakening effects of reinforcement. SF and PP fibers showed minimum resistance against post-peak cracking behavior. This may have been due to the less uniform dispersion of polypropylene fibers with SF. Nevertheless, all the beams with (5–10 and 15–20 mm) coarse aggregates exhibited deflection hardening behavior due to the higher dosage of steel fiber contents with polypropylene fibers. Some of them also showed multiple cracking behaviors. Multiple cracking was not observed in specimens of 15–20 mm size aggregates. The hybrid uses of polypropylene fibers, steel fibers, and nanocarbon black positively influenced the toughness parameters. Compared with monophasic conductive material, carbon black and polypropylene fibers significantly increased post-cracking behavior with steel fibers in both sizes of aggregates, as shown in Figure 3.

### 4.3. Effect of Different Admixtures and Coarse Aggregates Size on the Flexural Load-Bearing Capacity and (FCR)

**Figure 4:**
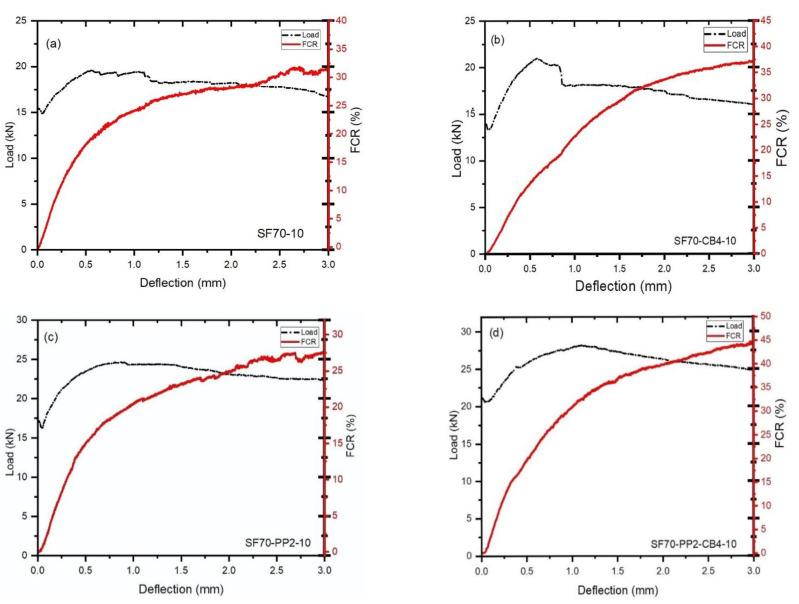
Load-deflection-FCR curves of 5–10 mm coarse aggregates (**a**) SF70-10 (**b**) SF70-CB4-10 (**c**) SF70-PP2-10 (**d**) SF70-PP2-CB4-10.

**Figure 5:**
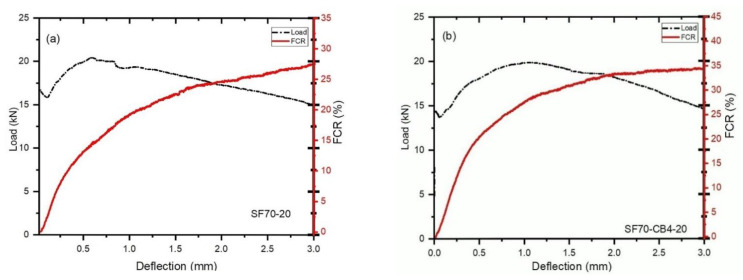

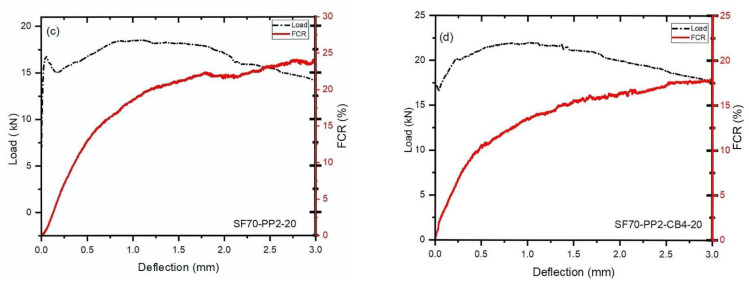
Load-deflection-FCR curves of 15–20 mm coarse aggregates (**a**) SF70-20 (**b**) SF70-CB4-20 (**c**) SF70-PP2-20 (**d**) SF70-PP2-CB4-20.

Figure 4a–d and Figure 5a–d show the difference between load, deflection and fraction change in resistance (FCR) with two different types of aggregates and fibers beams under bending. For the monophasic materials (SF70-10 and SF70-20), the signal-to-noise ratio was a little higher than that in diphasic and triphasic conductive and nonconductive materials. As shown in Figure 4b,d, the combined use of CB and SF had a positive influence and increased the signal-to-noise ratio of fractional change in resistance. During the first cracking, a significant increment of fractional change in resistance was observed. Abrupt loads decreased up to 17.48%, 8.33%, 5.32% and 2.70%, respectively, after the first cracking of SF70-10, SF70-CB4-10, SF70-PP2-10 and SF70-PP2-CB4-10 occurred. As shown in Figure 4b–d, the addition of carbon black with steel fiber and polypropylene fibers decreased the loading drop rate after first cracking and increased the percentage of FCR compared to SF70-10. The 20 mm size aggregates displayed greater loading drop rate ratios and low incremental rates of FCR compared to 10 mm size aggregates. In 20 mm aggregates, during the first cracking, abrupt loads were decreased up to 20.01%, 10.39%, 11.87%, and 12.71%, respectively. However, compared to SF-20, the loading drop rate observed was lower for other specimens, due to the inclusion of fibers. 

Compared with 10 mm aggregates, the first loading drop rate of 20 mm aggregates was much higher; up to 45.45%, 22.79%, 45.50%, and 55.55%, respectively. The load-bearing capacity of both sizes of aggregates improved slightly and then reduced gradually after the first load drop. The deflection softening and deflection hardening behaviors demonstrated the bending behavior of fiber-reinforced concrete. If the post-cracking behavior of a beam possesses a lower flexural load (as compared to cracking load), it is called deflection softening behavior. If the post-cracking behavior of a beam possesses higher flexural load than cracking load, it is called deflection hardening behavior [49,50].

Deflection hardening behavior was observed in all cases of both sizes of aggregates. This was observably due to the higher steel fiber content. The differences between the load-bearing capacity and the fractional change in resistance of fiber-reinforced concrete after the first cracking are described in Table 4. As shown in Figure 5a–d and Table 4, the loading drop rates were 1.50 kN, 1.05 kN, 0.97 kN and 0.60 kN from the first peak load of the samples SF70-10, SF70-CB4-10, SF70-PP2-10 and SF70-PP2-CB4-10, respectively. The change values of FCR at the deflection rate of 1.5 mm (FCR_1.5_) and 3 mm (FCR_3_) are described in Table 4. The (FCR_1.5_) of 10 mm size aggregates (SF70-10, SF70-CB4-10, SF70-PP2-10, SF70-PP2-CB4-10) increased (up to 25.25%, 28.95%, 23.56% and 35.12%, respectively), as did the values of (FCR_3_) (up to 31.9%, 37.52%, 27.65% and 44.95%, respectively). The increment of (FCR_3_) increased to 26.33%, 28.22%, 17.35%, and 21.86%, respectively, as compared to (FCR_1.5_) with the SF70-10, SF70-CB4-10, SF70-PP2-10 and SF70-PP2-CB4-10 samples, respectively. Compared to 20 mm aggregates, the (FCR_1.5_) of SF70-20, SF70-CB4-20, SF70-PP2-20 and SF70-PP2-CB4-20 increased to 21.15%, 30.25%, 20.20% and 15.18%, respectively, and the values of (FCR_3_) improved, reaching 28.12%, 35%, 24.15% and 17.10%, respectively.

The incremental rate of fractional change in resistance was higher in smaller aggregates than in larger aggregates. The incremental rate of (FCR_3_) for 10 mm aggregates with SF70-10, SF70-CB4-10, SF70-PP2-10 and SF70-PP2-CB4-10 increased 11.84%, 7%, 12.65%, and 66.22%, respectively, as compared to SF70-20, SF70-CB4-20, SF70-PP2-20 and SF70-PP2-CB4-20. Results indicated that the FCR values increased with the addition of carbon black with steel fibers at the time of deflection for both types of aggregates.

The addition of polypropylene fibers did not show any significant increment on FCR in aggregates with both sizes; however, as a triphasic material, the PP fibers with SF and CB showed higher increments in the case of smaller aggregates. This was due to the fact that a higher dosage of steel fibers provides more conductive fibers with lower volume content of nonconductive PP fibers, while carbon black creates an effective conductive path between the fiber network, which leads to an increase in the FCR. The greater size aggregates did not show higher FCR values because larger aggregates created large voids in concrete which disrupted the conductive paths. This could also have been attributable to the extensive gap between aggregates and the inhomogeneous distribution of fibers.

### 4.4. The Relationship between FCR and COD with Different Types of Admixtures and Coarse Aggregates

**Figure 6:**
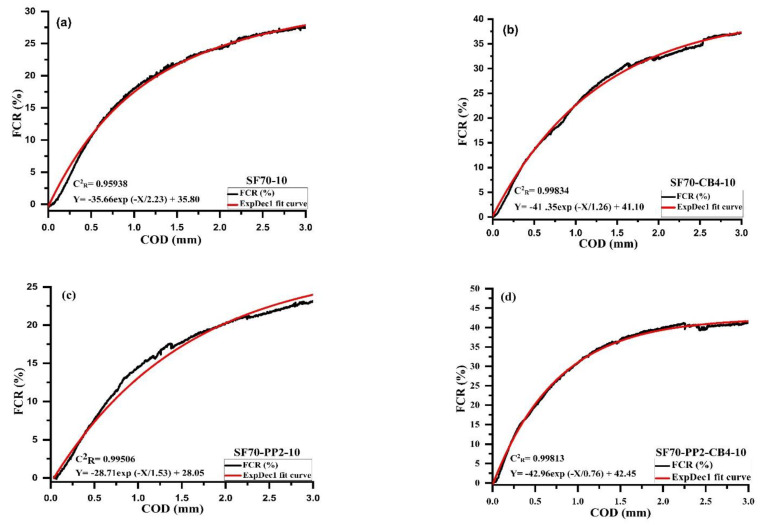
Relationship between FCR and COD of (**a**) SF70-10 (**b**) SF70-CB4-10 (**c**) SF70-PP2-10 (**d**) SF70-PP2-CB4-10.

**Figure 7:**
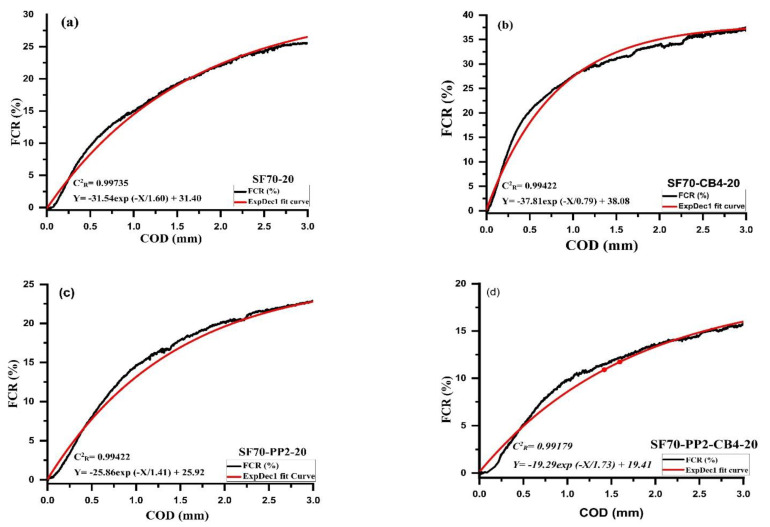
Relationship between FCR and COD of (**a**) SF70-20 (**b**) SF70-CB4-20 (**c**) SF70-PP2-20 (**d**) SF70-PP2-CB4-20.

The relationship between FCR and crack opening displacement (COD) with different types of conductive and nonconductive fibers contents on the tension side of beams under tension are illustrated in Figure 6a–d and Figure 7a–d. As can be seen from curves of aggregates with different sizes, the relationship between crack opening displacement and the fractional change in resistance was well associated with First Order Exponential Decay Function. The First Order Exponential Decay Function is determined by Equation (3).

*Y* = *A* × *exp* (−*X*/*t*) + *y*_0_ (3)

where *X* is the crack opening displacement measurement (mm), *Y* is the percentage of FCR_3_ which is measured at the distance of 3 mm, *t*, *y*_0_ and *A* are the constant parameters correlated to the type and amount of different admixture material, as described in Figure 6a–d and Figure 7a–d. Table 5 indicates the influence of different conductive admixtures with and without polypropylene fibers on the percentage of FCR_3_ values. The correlation coefficient C^2^_R_ for all the beams range varied from 0.959 to 0.998, as illustrated in Table 5.

It showed a strong relationship with Equation (3) between the fraction change in resistance and crack opening displacement. The samples containing 10 mm size aggregates with SF70-10 kg/m^3^ steel fibers were compared with diphasic and triphasic admixtures. Relative to monophasic (SF70-10) and (SF70-PP2-10), the FCR_3_ values of diphasic and triphasic conductive admixtures increased. The values of FCR_3_ increased up to 27.75%, 38.95%, 23.65% and 42.50%, respectively, for SF70-10, SF70-CB4-10, SF70-PP2-10 and SF70-PP2-CB4-10. The value of SF70-CB4-10 and SF70-PP2-CB4-10 was higher due to the addition of carbon with steel fiber and PP fibers. Compared with SF70-10, the FCR_3_ values of SF70-CB4-10 and SF70-PP2-CB4-10 improved by 28.75% and 34.70%, respectively.

For the specimens containing 20 mm aggregates with SF70-20 steel fiber content, the values of FCR_3_ increased with the addition of carbon black. However, these values were not higher than those of 10 mm aggregate specimens. The values of FCR_3_ for all 20 mm size specimens increased by 26.5%, 35%, 22.50% and 16.50%, respectively. The FCR_3_ values increased by 39.62% and 35.84%, respectively for SF70-CB4-20 and SF70-PP2-CB4-20 when compared to SF70-20. As compared to 10 mm aggregate specimens (SF70-10, SF70-CB4-10, SF70-PP2-10 and SF70-PP2-CB4-10), the values of 20 mm aggregates (SF70-20, SF70-CB4-20, SF70-PP2-20, SF70-PP2-CB4-20) decreased by 4.5%, 10.32%, 4.12% and 61%, respectively. In both aggregate sizes, the FCR_3_ increment was observed, with an addition of carbon black in diphasic and triphasic admixtures. The NCB particles were devoted to different fibers in the form of grapes [51]. The nanocarbon black with fibers acted similarly to a bunch of grapes, which improved the effective conductive path.

### 4.5. Multiple Cracking Monitoring

**Figure 8:**
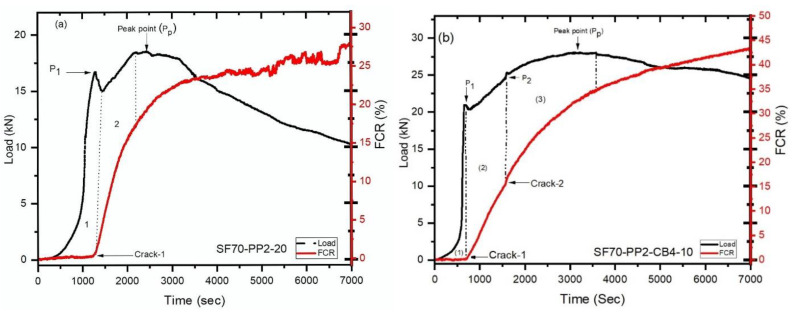
Load-time-FCR curves of (**a**) SF70-PP2-20 (**b**) SF70-PP2-CB4-10.

**Figure 9:**
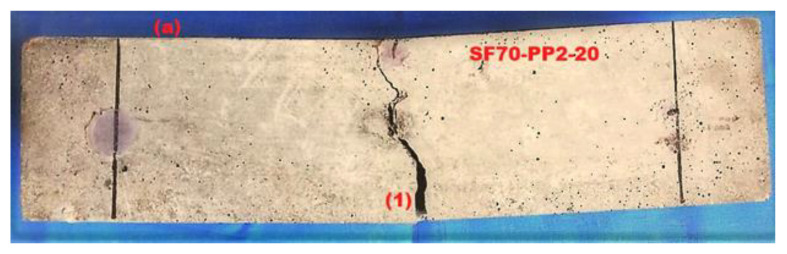

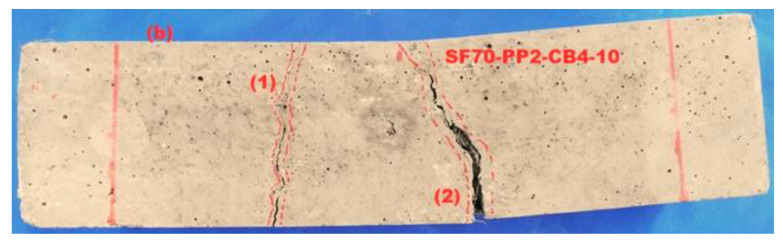
Single and multiple crack patterns of (**a**) SF70-PP2-20 (**b**) SF70-PP2-CB4-10.

The relationship between different beams with conductive and nonconductive fibers was analyzed under bending. The correlation of load-time-FCR and the law of resistance signals variances from all beams was similar with different coarse aggregates and different fibers. This research presented an experimental investigation of crack opening and its propagation precisely with fractional changes in resistance in fiber-reinforced concrete beams. As described in our explanation of multiple cracking, there was a nonlinearity in a load-time curve—that is, the first cracking load [52]. At the same time, as the load dropped, the FCR began increasing, leading to the appearance of the first crack. The increase of FCR with the first crack was due to the discontinuation of the conductive path, which was produced and maintained by triphasic conductive admixtures. After that nonlinearity, the load increased to some extent. After reaching a certain height, the load dropped again, synchronically. A clear change in FCR increasing rate was observed in the FCR-time curve, evidence of the second crack. If a load was dropped with significant force, a straight vertical line formed related to the magnitude of that dropped load. If the load drop was not significant, it simply caused a change in the rate of FCR increase. For single and multiple cracking, two beams were presented separately. One beam was taken from (5–10 mm) size (SF70-PP2-CB4-10) coarse aggregates, which showed multiple cracking behaviors, and another was taken from (15–20 mm) coarse aggregate (SF70-PP2-20), which demonstrated single cracking behavior. All beams of (15–20 mm) coarse aggregates failed to show multiple cracking behaviors. Figure 8a,b indicates the load-time-fractional change in resistance for two different sizes of coarse aggregates (SF70-PP2-20) and (SF70-PP2-CB4-10) specimens. The left and the right vertical axis of Figure 8a,b demonstrate the load and FCR, while the horizontal axis represents time. The load-time relationship, represented with the solid line and dotted line curves, also demonstrates the time-FCR relationship. After first cracking, the concrete beams showed two behaviors: deflection softening and deflection hardening under load-deflection curves. Deflection hardening behavior means the beam showed a higher load-carrying capacity after the first cracking. If the first cracking load (P_i_) and peak load (P_P_) were equivalent to each other, that is deflection softening behavior, and if the peak load (P_p_) is greater than that of the first cracking load (P_i_), it is called deflection hardening behavior [15]. Figure 8a is split into two sections; the pre-cracking region is shown in part 1 and the post-cracking region of the concrete beam (SF70-PP2-20) is shown in part 2. The deflection hardening behavior can be seen in the post-cracking region, as peak load (P_p_) was greater than that of the first cracking load (P_i_). The initial load linearly increased up to 16.75 kN (P_i_) and then suddenly dropped to 14.95 kN, as shown in Figure 8a. The fractional change in resistance led to an increase after the initial crack due to the discontinuance of the conductive path after the first crack formed by diphasic material. According to the FCR-time curve, the fractional change in resistance was enhanced parabolically after the crack widening increased as far as the load starting drop. The first load decline is illustrated as a vertical straight dotted line by the FCR-time curve. After the first cracking, an increment was observed up to 18.60 kN, higher than the first dropping load. As the load in the post-cracking area increased, the FCR also increased continuously. This increment can be attributed to the steel fiber and polypropylene fiber withstanding the larger load after first cracking and restricting greater propagation and formation of cracks. The single crack was observed with deflection hardening behavior in the concrete member, as shown in Figure 9a. The point where the nonlinearity appears in the load-deflection curve after initial cracking is called the first cracking point.

The same phenomenon was observed in the case of Figure 8b, which has indicated the multiple cracking behaviour. In part 1 of Figure 9b, after the loading drop rate of (P_i_) 20.35 kN, the increment of FCR is noticed. During the first drop down of load, the fractional change in resistance gives the signs to increase the (FCR) which clearly exhibited the initial cracking.

After the appearance of the initial crack (P_i_), the peak load (P_2_) increased further, up to 24.45 kN. As shown in Part 2 of Figure 9b, from the first crack (Pi) to the second crack (P_2_), a linear increment was observed in the FCR-time curve and in the load-time curve. The loading drop rate was very low at this point, but then propagated gradually. After the second cracking, it was clearly observable that the (FCR) changed the resistance with the time-FCR curve, which is a sign of multiple cracking. This was also noted during the visual inspection of the testing process, as depicted in Figure 9b. The FCR-time is represented by a straight, dotted, vertically increasing line which indicates the loading drop rate (P_2_). The load increased again, gradually, up to (P_p_) 28.20 kN, after displaying the second crack and, at the same time, the time-FCR curve enhanced exponentially from (P_2_) to peak load (P_p_). The FCR-time curve (in the form of a parabola) increased until the load reached its peak, which is shown in part 3 of Figure 9b.

During these experiments, cracks were monitored using different aggregates with conductive and nonconductive fibers. Nonetheless, steel fiber and carbon black were more capable of monitoring the crack position in all types of concrete beams with smaller aggregates.

## 5. Discussion

The effects of hybridization of steel fiber and pp fiber, along with filler material (carbon black) were studied in the present work. The effects of the combined use of fibers exhibitied a positive effect on the post-cracking behavior of the beam. The smaller coarse aggregates improved compressive strength, attributable to the fact they reduced the volume of voids within the material and improved the microstructure and interfacial transition zone (ITZ), thus reducing the crack initiation in the matrix. The bond strength between the aggregate and the matrix also improved, which ultimately led to improved compressive strength of the matrix and higher load-deflection behavior. The smaller coarse aggregates reduced the number of videos as compared to the greater number of coarse aggregates. Additionally, as carbon black fills voids and improves the microstructure and the bond between fibers, it ultimately improves the flexural toughness parameters.

## 6. Conclusions

This study evaluated self-sensing behaviors in reinforced concrete, incorporating different-sized aggregates (5–10 mm and 15–20 mm) and fibers (steel fibers, polypropylene fibers, carbon black) under a four-point bending test. The mechanical properties, post-cracking behavior, toughness parameters, conductive performance, and self-sensing ability of different sizes of aggregates with different fibers were investigated. One potential application could be in early detection of cracks in bridge girders and slabs, which could prevent the damages to structures and dangers to human lives, in addition to reducing retrofitting costs at later stages. The following conclusions were drawn from from the research findings:

1. The addition of steel fibers and carbon black (monophasic and diphasic) increased the compression strength of both sizes of aggregates as compared to plain concrete. However, the inclusion of polypropylene fibers as a triphasic material (SF + PP + CB) led to a minor reduction in compression strength in different sizes of aggregates.
2. The hybridization of steel fibers with carbon black and polypropylene fibers can improve the flexural strength of both sizes of aggregates. Smaller aggregates (5–10 mm) showed a higher flexural strength ratio than larger size aggregates (15–20 mm).
3. The post-cracking behaviour and toughness parameters of specimens were significantly improved by the addition of steel fiber and polypropylene fiber. Smaller aggregates demonstrated higher energy absorption capacity and flexural toughness compared to larger ones.
4. The diphasic and triphasic conductive material with smaller aggregates (5–10 mm) increased FCR values up to 38.95% and 42.21%, respectively, for SF70-CB4-10 and SF70-PP2-CB4-10 admixtures; with larger aggregates, the FCR values increased up to 35.5% and 16.50%, respectively, for SF70-CB4-20 and SF70-PP2-CB4-20 admixtures.
5. The hybrid use of polypropylene fibers with steel fibers decreased the (FCR) values, but as triphasic material (SF + PP + CB), they increased the (FCR) values due to the conductive characteristics of carbon black.
6. Multiple cracking can be sensed by the load-time-FCR graph, as the linear increment of fractional change in resistance (FCR) was noted during the first peak load, followed by an observable clear variation in FCR after the occurrence of the second crack.

## Figures and Tables

**Table 1:** Physical properties of fiber and filler materials.

Materials	Length (mm)	Diameter/Particle Size (mm)	Density (g/cm^3^)	Resistivity (Ω·cm)	Tensile Strength (MPa)	Young’s Modulus (GPa)
SF	35	0.55	7.85	5–10	1150	210
PP	35	0.35	0.9	-	547–658	3.5–7.5
CB	-	6 × 10^−5^	0.5	0.75	-	-

**Table 2:** Mix proportions of concrete with conductive and nonconductive material.

Type	Specimens	Binder (kg/m^3^)	Aggregates (kg/m^3^)	Polypropylene Fiber (PP) (kg/m^3^)	Steel Fiber (SF) (kg/m^3^)	Carbon Black (CB) (kg/m^3^)	Slump (mm)
Plain Concrete	PC-10	545	1670	-	-	-	180
	PC-20	545	1670	-	-	-	195
Monophasic admixtures	SF70-10	545	1670	-	70	-	153
	SF70-20	545	1670	-	70	-	164
Diphasic Admixtures	SF70-CB4-10	545	1670	-	70	4	69
	SF70-CB4-20	545	1670	-	70	4	81
	SF70-PP2-10	545	1670	2	70	-	150
	SF70-PP2-20	545	1670	2	70	-	162
Triphasic Admixtures	SF70-PP2-CB4-10	545	1670	2	70	4	63
	SF70-PP2-CB4-20	545	1670	2	70	4	71

Notations: PC-10 and PC-20 stands for plain concrete with 5–10 mm and 15–20 mm size coarse aggregates. SF, PP and CB stand for steel fiber, polypropylene fiber and carbon black. The number followed by the letter refers to the conductive material size, kg/m^3^, for example, SF70-PP2-CB4-10 and SF70-PP2-CB4-20 mean the samples with steel fibers 70 kg/m^3^, polypropylene fiber 2 kg/m^3^, carbon black 4 kg/m^3^ and coarse aggregates with (5–10 mm) and (15–20 mm) size aggregates.

**Table 3:** Compression strength, flexural strength and toughness parameters.

Specimens	*f_cu_* (N/mm^2^)	Standred Deviation	F_u_ (kN)	σ_u_ (N/mm^2^)	*D^f^_BZ_*._2_	*f_eq_*_.2_	*D^f^_BZ_*._3_	*f_eq_*_.3_
PC-10	40.27	3.83	13.50	4.05	-	-	-	-
PC-20	37.76	4.95	12.94	3.88	-	-	-	-
SF70-10	43.01	1.57	15.88	4.76	8.44	7.60	46.232	8.33
SF70-20	40.31	2.37	15.10	4.53	9.53	8.59	44.19	7.96
SF70-CB4-10	46.04	1.91	17.18	5.15	10.83	9.70	54.11	10.10
SF70-CB4-20	42.21	3.73	14.59	4.37	8.60	7.74	45.42	8.18
SF70-PP2-10	38.65	3.72	16.60	5.04	11.49	10.34	58.87	11.21
SF70-PP2-20	35.55	3.07	15.88	4.76	8.28	7.50	43.01	7.74
SF70-PP2-CB4-10	41.47	6.51	20.97	6.04	12.10	10.90	66.17	11.97
SF70-PP2-CB4-20	38.33	7.83	16.62	4.96	11.17	10.23	53.45	9.62

Notations, *f_cu_*: compressive strength of concrete (N/mm^2^), F_u_: the values of maximum load (kN) at the interval of 0.05 mm, σ_u_: flexural strength of concrete (N/mm^2^), *f_eq_*_.2_: equivalent flexural tensile strength (Mpa) by δ_2_, *f_eq_*_.3_: equivalent flexural tensile strength (Mpa) by δ_3_, *D^b^_BZ_*: The energy absorption capacity of plain concrete, *D^f^_BZ_*._2_ = *D_BZ_*_2_ − *D^b^_BZ_* (kN·mm) by δ_2_ = δ_1_ + 0.65 (mm), *D^f^_BZ_*._3_ = *D_BZ_*_3_ − *D^b^_BZ_* (kN·mm) by δ_3_ = δ_1_ + 2.65 (mm), δ: deflection corresponds to F_u_ (mm) described in Figure 3.

**Table 4:** Loading drop rate and percentage increment of FCR.

Samples	Load Drop Rate (%)	Load Drop (kN)	FCR_1.5_ (%)	FCR_3_ (%)	Percentage Increment from FCR_1.5_ to FCR_3_
SF70-10	17.48	1.50	25.25	31.9	20.84
SF70-20	20.01	2.75	21.15	28.12	24.50
SF70-CB4-10	8.33	1.05	28.95	37.72	23.25
SF70-CB4-20	10.39	1.36	28.25	34.5	18.57
SF70-PP2-10	5.32	0.97	23.56	27.65	14.79
SF70-PP2-20	11.87	1.80	20.20	24.15	16.35
SF70-PP2-CB4-10	2.70	0.60	35.12	44.95	21.86
SF70-PP2-CB4-10	12.71%	1.35	15.18	17.10	11.22

**Table 5:** Fitted parameters through exp decay fit equation.

Specimens	*t*	*A*	*Y*_0_	C^2^_R_	FCR_3_ (%)
SF70-10	2.23	−35.66	35.80	0.95938	27.75
SF70-20	1.60	−31.54	31.40	0.99735	26.12
SF70-CB4-10	1.26	−41.35	41.10	0.99834	38.95
SF70-CB4-20	0.79	−37.81	38.08	0.99422	35
SF70-PP2-10	1.53	−28.71	28.05	0.99506	23.65
SF70-PP2-20	1.41	−25.86	25.92	0.99422	22.50
SF70-PP2-CB4-10	0.76	−42.96	42.45	0.99813	42.50
SF70-PP2-CB4-20	1.73	−19.29	19.41	0.99179	16.50

## Data Availability

The data presented in this article are available within the article.
